# Supplementary material for: Modelling Skylarks (Alauda arvensis) to Predict Impacts of Changes in Land Management and Policy: Development and Testing of an Agent-Based Model
Source: PLoS One. 2013 Jun 6;8(6):e65803. doi: 10.1371/journal.pone.0065803 (PMC3675089; doi:10.1371/journal.pone.0065803)
Supplement: Supporting Information S4 — The skylark ODdox as a zipped archive. (ZIP) [file pone.0065803.s004.zip › Skylark_ODdox/globals_0x74.html]

ALMaSS Skylark ODdox: File Members


ALMaSS Skylark ODdox
 2.0


- Main Page
- Related Pages
- Classes
- Files

- File List
- File Members

- All
- Functions
- Variables
- Typedefs
- Enumerations
- Enumerator
- Macros

- \_
- a
- b
- c
- d
- e
- f
- g
- h
- i
- j
- k
- l
- m
- n
- o
- p
- r
- s
- t
- u
- v
- w
- x

Here is a list of all file members with links to the files they belong to:

### - t -

- tbl\_country
  : Rodenticide.h
- tbl\_foobar
  : Rodenticide.h
- tbl\_town
  : Rodenticide.h
- tbl\_woodland
  : Rodenticide.h
- TempHindrance
  : skylarks\_all.cpp
- TerrHeterogeneity
  : skylarks\_all.cpp
- terrnos
  : skylarks\_all.cpp
- terrsize
  : skylarks\_all.cpp
- TLANDSCAPE\_H
  : landscape.h
- TListOfAnimals
  : populationmanager.h
- tof\_AgroChemIndustryCerealFarm1
  : farm.h
- tof\_AgroChemIndustryCerealFarm2
  : farm.h
- tof\_AgroChemIndustryCerealFarm3
  : farm.h
- tof\_ConventionalCattle
  : farm.h
- tof\_ConventionalPig
  : farm.h
- tof\_ConventionalPlant
  : farm.h
- tof\_ConvMarginalJord
  : farm.h
- tof\_NoPesticideBase
  : farm.h
- tof\_NoPesticideNoP
  : farm.h
- tof\_OrganicCattle
  : farm.h
- tof\_OrganicPig
  : farm.h
- tof\_OrganicPlant
  : farm.h
- tof\_PTrialControl
  : farm.h
- tof\_PTrialToxicControl
  : farm.h
- tof\_PTrialTreatment
  : farm.h
- tof\_UserDefinedFarm1
  : farm.h
- tof\_UserDefinedFarm10
  : farm.h
- tof\_UserDefinedFarm11
  : farm.h
- tof\_UserDefinedFarm12
  : farm.h
- tof\_UserDefinedFarm13
  : farm.h
- tof\_UserDefinedFarm14
  : farm.h
- tof\_UserDefinedFarm15
  : farm.h
- tof\_UserDefinedFarm16
  : farm.h
- tof\_UserDefinedFarm17
  : farm.h
- tof\_UserDefinedFarm18
  : farm.h
- tof\_UserDefinedFarm19
  : farm.h
- tof\_UserDefinedFarm2
  : farm.h
- tof\_UserDefinedFarm20
  : farm.h
- tof\_UserDefinedFarm21
  : farm.h
- tof\_UserDefinedFarm22
  : farm.h
- tof\_UserDefinedFarm23
  : farm.h
- tof\_UserDefinedFarm24
  : farm.h
- tof\_UserDefinedFarm25
  : farm.h
- tof\_UserDefinedFarm26
  : farm.h
- tof\_UserDefinedFarm27
  : farm.h
- tof\_UserDefinedFarm28
  : farm.h
- tof\_UserDefinedFarm29
  : farm.h
- tof\_UserDefinedFarm3
  : farm.h
- tof\_UserDefinedFarm30
  : farm.h
- tof\_UserDefinedFarm31
  : farm.h
- tof\_UserDefinedFarm32
  : farm.h
- tof\_UserDefinedFarm33
  : farm.h
- tof\_UserDefinedFarm34
  : farm.h
- tof\_UserDefinedFarm35
  : farm.h
- tof\_UserDefinedFarm4
  : farm.h
- tof\_UserDefinedFarm5
  : farm.h
- tof\_UserDefinedFarm6
  : farm.h
- tof\_UserDefinedFarm7
  : farm.h
- tof\_UserDefinedFarm8
  : farm.h
- tof\_UserDefinedFarm9
  : farm.h
- tole\_ActivePit
  : tole\_declaration.h
- tole\_AmenityGrass
  : tole\_declaration.h
- tole\_BareRock
  : tole\_declaration.h
- tole\_BeetleBank
  : tole\_declaration.h
- tole\_Building
  : tole\_declaration.h
- tole\_BuiltUpWithParkland
  : tole\_declaration.h
- tole\_Coast
  : tole\_declaration.h
- tole\_ConiferousForest
  : tole\_declaration.h
- tole\_Copse
  : tole\_declaration.h
- tole\_DeciduousForest
  : tole\_declaration.h
- tole\_Field
  : tole\_declaration.h
- tole\_FieldBoundary
  : tole\_declaration.h
- tole\_Foobar
  : tole\_declaration.h
- tole\_Freshwater
  : tole\_declaration.h
- tole\_Garden
  : tole\_declaration.h
- tole\_Heath
  : tole\_declaration.h
- tole\_HedgeBank
  : tole\_declaration.h
- tole\_Hedges
  : tole\_declaration.h
- tole\_LargeRoad
  : tole\_declaration.h
- tole\_Marsh
  : tole\_declaration.h
- tole\_MixedForest
  : tole\_declaration.h
- tole\_NaturalGrass
  : tole\_declaration.h
- tole\_Orchard
  : tole\_declaration.h
- tole\_OrchardBand
  : tole\_declaration.h
- tole\_OrchardGrass
  : tole\_declaration.h
- tole\_Parkland
  : tole\_declaration.h
- tole\_PermanentSetaside
  : tole\_declaration.h
- tole\_PermPasture
  : tole\_declaration.h
- tole\_PermPastureLowYield
  : tole\_declaration.h
- tole\_PermPastureTussocky
  : tole\_declaration.h
- tole\_PitDisused
  : tole\_declaration.h
- tole\_Railway
  : tole\_declaration.h
- tole\_River
  : tole\_declaration.h
- tole\_RiversidePlants
  : tole\_declaration.h
- tole\_RiversideTrees
  : tole\_declaration.h
- tole\_RoadsideVerge
  : tole\_declaration.h
- tole\_RuralResidential
  : tole\_declaration.h
- tole\_Saltwater
  : tole\_declaration.h
- tole\_SandDune
  : tole\_declaration.h
- tole\_Scrub
  : tole\_declaration.h
- tole\_SmallRoad
  : tole\_declaration.h
- tole\_StoneWall
  : tole\_declaration.h
- tole\_Suburban
  : tole\_declaration.h
- tole\_Track
  : tole\_declaration.h
- tole\_UnsprayedFieldMargin
  : tole\_declaration.h
- tole\_Urban
  : tole\_declaration.h
- tole\_UrbanNoVeg
  : tole\_declaration.h
- tole\_UrbanPark
  : tole\_declaration.h
- tole\_YoungForest
  : tole\_declaration.h
- toss\_AttractingAMate
  : skylarks\_all.h
- toss\_BuildingUpResources
  : skylarks\_all.h
- toss\_CDying
  : skylarks\_all.h
- toss\_Destroy
  : skylarks\_all.h
- toss\_Developing
  : skylarks\_all.h
- toss\_EggHatching
  : skylarks\_all.h
- toss\_FArriving
  : skylarks\_all.h
- toss\_FCaringForYoung
  : skylarks\_all.h
- toss\_FDying
  : skylarks\_all.h
- toss\_FEmigrating
  : skylarks\_all.h
- toss\_FFindingTerritory
  : skylarks\_all.h
- toss\_FFloating
  : skylarks\_all.h
- toss\_FFlocking
  : skylarks\_all.h
- toss\_FImmigrating
  : skylarks\_all.h
- toss\_FollowingMate
  : skylarks\_all.h
- toss\_FTempLeavingArea
  : skylarks\_all.h
- toss\_GivingUpTerritory
  : skylarks\_all.h
- toss\_Hatching
  : skylarks\_all.h
- toss\_Incubating
  : skylarks\_all.h
- toss\_Initiation
  : skylarks\_all.h
- toss\_Laying
  : skylarks\_all.h
- toss\_MakingNest
  : skylarks\_all.h
- toss\_MArriving
  : skylarks\_all.h
- toss\_MCaringForYoung
  : skylarks\_all.h
- toss\_MDying
  : skylarks\_all.h
- toss\_MEmigrating
  : skylarks\_all.h
- toss\_MFindingTerritory
  : skylarks\_all.h
- toss\_MFloating
  : skylarks\_all.h
- toss\_MFlocking
  : skylarks\_all.h
- toss\_MImmigrating
  : skylarks\_all.h
- toss\_MRehousing
  : skylarks\_all.h
- toss\_MTempLeavingArea
  : skylarks\_all.h
- toss\_NDeveloping
  : skylarks\_all.h
- toss\_NDying
  : skylarks\_all.h
- toss\_NMaturing
  : skylarks\_all.h
- toss\_PDeveloping
  : skylarks\_all.h
- toss\_PDying
  : skylarks\_all.h
- toss\_PMaturing
  : skylarks\_all.h
- toss\_PreparingForBreeding
  : skylarks\_all.h
- toss\_ScaringOffChicks
  : skylarks\_all.h
- toss\_StartingNewBrood
  : skylarks\_all.h
- toss\_StoppingBreeding
  : skylarks\_all.h
- tov\_AgroChemIndustryCereal
  : tov\_declaration.h
- tov\_Carrots
  : tov\_declaration.h
- tov\_CloverGrassGrazed1
  : tov\_declaration.h
- tov\_CloverGrassGrazed2
  : tov\_declaration.h
- tov\_FieldPeas
  : tov\_declaration.h
- tov\_FieldPeasStrigling
  : tov\_declaration.h
- tov\_FodderBeet
  : tov\_declaration.h
- tov\_FodderGrass
  : tov\_declaration.h
- tov\_Lawn
  : tov\_declaration.h
- tov\_Maize
  : tov\_declaration.h
- tov\_MaizeSilage
  : tov\_declaration.h
- tov\_MaizeStrigling
  : tov\_declaration.h
- tov\_NaturalGrass
  : tov\_declaration.h
- tov\_NoGrowth
  : tov\_declaration.h
- tov\_None
  : tov\_declaration.h
- tov\_Oats
  : tov\_declaration.h
- tov\_OBarleyPeaCloverGrass
  : tov\_declaration.h
- tov\_OCarrots
  : tov\_declaration.h
- tov\_OCloverGrassGrazed1
  : tov\_declaration.h
- tov\_OCloverGrassGrazed2
  : tov\_declaration.h
- tov\_OCloverGrassSilage1
  : tov\_declaration.h
- tov\_OFieldPeas
  : tov\_declaration.h
- tov\_OFieldPeasSilage
  : tov\_declaration.h
- tov\_OFirstYearDanger
  : tov\_declaration.h
- tov\_OGrazingPigs
  : tov\_declaration.h
- tov\_OMaizeSilage
  : tov\_declaration.h
- tov\_OOats
  : tov\_declaration.h
- tov\_OPermanentGrassGrazed
  : tov\_declaration.h
- tov\_OPotatoes
  : tov\_declaration.h
- tov\_Orchard
  : tov\_declaration.h
- tov\_OSBarleySilage
  : tov\_declaration.h
- tov\_OSeedGrass1
  : tov\_declaration.h
- tov\_OSeedGrass2
  : tov\_declaration.h
- tov\_OSetaside
  : tov\_declaration.h
- tov\_OSpringBarley
  : tov\_declaration.h
- tov\_OSpringBarleyClover
  : tov\_declaration.h
- tov\_OSpringBarleyExt
  : tov\_declaration.h
- tov\_OSpringBarleyGrass
  : tov\_declaration.h
- tov\_OSpringBarleyPigs
  : tov\_declaration.h
- tov\_OTriticale
  : tov\_declaration.h
- tov\_OWinterBarley
  : tov\_declaration.h
- tov\_OWinterBarleyExt
  : tov\_declaration.h
- tov\_OWinterRape
  : tov\_declaration.h
- tov\_OWinterRye
  : tov\_declaration.h
- tov\_OWinterWheatUndersown
  : tov\_declaration.h
- tov\_OWinterWheatUndersownExt
  : tov\_declaration.h
- tov\_PermanentGrassGrazed
  : tov\_declaration.h
- tov\_PermanentGrassLowYield
  : tov\_declaration.h
- tov\_PermanentGrassTussocky
  : tov\_declaration.h
- tov\_PermanentSetaside
  : tov\_declaration.h
- tov\_Potatoes
  : tov\_declaration.h
- tov\_PotatoesIndustry
  : tov\_declaration.h
- tov\_SeedGrass1
  : tov\_declaration.h
- tov\_SeedGrass2
  : tov\_declaration.h
- tov\_Setaside
  : tov\_declaration.h
- tov\_SpringBarley
  : tov\_declaration.h
- tov\_SpringBarleyCloverGrass
  : tov\_declaration.h
- tov\_SpringBarleyCloverGrassStrigling
  : tov\_declaration.h
- tov\_SpringBarleyGrass
  : tov\_declaration.h
- tov\_SpringBarleyPeaCloverGrassStrigling
  : tov\_declaration.h
- tov\_SpringBarleyPTreatment
  : tov\_declaration.h
- tov\_SpringBarleySeed
  : tov\_declaration.h
- tov\_SpringBarleySilage
  : tov\_declaration.h
- tov\_SpringBarleySKManagement
  : tov\_declaration.h
- tov\_SpringBarleyStrigling
  : tov\_declaration.h
- tov\_SpringBarleyStriglingCulm
  : tov\_declaration.h
- tov\_SpringBarleyStriglingSingle
  : tov\_declaration.h
- tov\_SpringRape
  : tov\_declaration.h
- tov\_SpringWheat
  : tov\_declaration.h
- tov\_Triticale
  : tov\_declaration.h
- tov\_Undefined
  : tov\_declaration.h
- tov\_WinterBarley
  : tov\_declaration.h
- tov\_WinterBarleyStrigling
  : tov\_declaration.h
- tov\_WinterRape
  : tov\_declaration.h
- tov\_WinterRapeStrigling
  : tov\_declaration.h
- tov\_WinterRye
  : tov\_declaration.h
- tov\_WinterRyeStrigling
  : tov\_declaration.h
- tov\_WinterWheat
  : tov\_declaration.h
- tov\_WinterWheatShort
  : tov\_declaration.h
- tov\_WinterWheatStrigling
  : tov\_declaration.h
- tov\_WinterWheatStriglingCulm
  : tov\_declaration.h
- tov\_WinterWheatStriglingSingle
  : tov\_declaration.h
- tov\_WWheatPControl
  : tov\_declaration.h
- tov\_WWheatPToxicControl
  : tov\_declaration.h
- tov\_WWheatPTreatment
  : tov\_declaration.h
- tov\_YoungForest
  : tov\_declaration.h
- TramlinePremium
  : skylarks\_all.cpp
- trial\_control
  : treatment.h
- trial\_insecticidetreat
  : treatment.h
- trial\_toxiccontrol
  : treatment.h
- ttop\_EndocrineDisruptor
  : landscape.h
- ttop\_GeneralOrganophosphate
  : landscape.h
- ttop\_ModelinkPesticide
  : landscape.h
- ttop\_ModelinkPesticide21TWA
  : landscape.h
- ttop\_NoPesticide
  : landscape.h
- ttop\_Vinclozolin
  : landscape.h
- TTypesBaitLocation
  : Rodenticide.h
- TTypesOfFarm
  : farm.h
- TTypesOfLandscapeElement
  : tole\_declaration.h
- TTypesOfPesticide
  : landscape.h
- TTypesOfSkState
  : skylarks\_all.h
- TTypesOfVegetation
  : tov\_declaration.h


- Generated on Thu Jan 10 2013 13:15:39 for ALMaSS Skylark ODdox by
   1.8.1.1
